# A typology of nonsuicidal self‐injury in a clinical sample: A latent class analysis

**DOI:** 10.1002/cpp.2463

**Published:** 2020-05-20

**Authors:** Shazana Shahwan, Jue Hua Lau, Edimansyah Abdin, Yunjue Zhang, Rajeswari Sambasivam, Wen Lin Teh, Bhanu Gupta, Say How Ong, Siow Ann Chong, Mythily Subramaniam

**Affiliations:** ^1^ Research Division Institute of Mental Health Singapore; ^2^ Department of Mood and Anxiety Institute of Mental Health Singapore; ^3^ Department of Developmental Psychiatry Institute of Mental Health Singapore

**Keywords:** clinical, self‐harm, latent class, self‐injury, subtypes

## Abstract

Nonsuicidal self‐injury(NSSI) is a behavioural concern and can present in diverse ways, varying by method, frequency, severity, function and so forth. The possible combinations of these features of NSSI produce an array of profiles that makes evaluation and management of this behaviour challenging. The aim of this study was to build upon previous work that reduces the heterogeneity of NSSI patterns by using latent class analysis (LCA) to identify a typology of NSSI. Participants consisted of 235 outpatients aged 14–35 years attending a tertiary psychiatric hospital in Singapore who had reported at least one NSSI behaviour within the last year. Eight indicators captured using the Functional Assessment of Self‐Mutilation were used in the LCA: frequency of NSSI, length of contemplation before engaging in NSSI, usage of more than three NSSI methods, suicidal ideation and four psychological functions of NSSI, that is, social‐positive, social‐negative, automatic‐positive and automatic‐negative. The LCA revealed three distinct groups: Class 1—Experimental/Mild NSSI, Class 2—Multiple functions NSSI/Low Suicide Ideation and Class 3—Multiplefunctions NSSI/Possible Suicide Ideation. Multinomial logistic regression analyses were conducted to examine the associations between class membership and sociodemographic variables as well as measures of emotion dysregulation, childhood trauma, depression and quality of life. Females were overrepresented in Class 3. In general, Class 3 had the poorest scores followed by Class 2. Our analyses suggest that different NSSI subtypes require different treatment indications. Profiling patterns of NSSI may be a potentially useful step in guiding treatment plans and strategies.

**Key Practitioner Message**

- Nonsuicidal self‐injury (NSSI) is a strong predictor of suicide, though only minority of individuals who self‐injure harbour suicidal intent. Patterns of NSSI can also be differentiated using indicators such as modality, frequency and psychological function.
- Three groups of individuals who self‐injure were identified using latent class analysis: Class 1—Mild/Experimental, Class 2—Multiple functions NSSI/Low Suicide Ideation and Class 3—Multiple functions/Possible Suicide Ideation.
- These three classes have varying therapeutic needs. Thus, evaluating NSSI behaviour using such classification systems may be useful in guiding treatment plans and resource allocation.

## 1 INTRODUCTION

Nonsuicidal self‐injury (NSSI) refers to the direct and deliberate destruction or alteration of bodily tissue in the absence of suicidal intent (Nock & Favazza, 2009). Common forms of NSSI include cutting, hitting, burning, scratching, biting and interfering with wound healing (Nock & Favazza, 2009). Once regarded as a symptom of borderline personality disorder, researchers and various organizations have advocated for the recognition of NSSI as an independent disorder in future editions of the Diagnostic and Statistical Manual of Mental Disorders (DSM). However, due to the poor reliability of the NSSI disorder criteria in clinical and field trials, NSSI appears instead in the section ‘Conditions for Further Study' in the DSM‐Fifth Edition as a distinct syndrome (Gratz, Dixon‐Gordon, Chapman, & Tull, 2015; Zetterqvist, 2015). It is included as a condition ‘on which future research is encouraged', which is ‘not intended for clinical use' (Plener & Fegert, 2015). This criterion has led to some agreement on how NSSI is defined and growing convergence in prevalence rates across studies. NSSI rates have been reported to be 1–4% in the adult general population, 15–35% among adolescents and young adults and 40–80% among psychiatric patients (Kerr, Muehlenkamp, & Turner, 2010).

The stereotypical self‐injurer is often depicted as a young female who self‐injures by cutting herself (Whitlock & Eckenrode, 2008) and does so to seek attention (Timson, Priest, & Clark‐Carter, 2012). However, a number of studies have shown that males are just as likely to self‐injure as females though the former are inclined towards different forms such as self‐battery(Whitlock & Eckenrode, 2008). Although the motivation to influence others (e.g., elicit care and attention from significant others) may be a function of NSSI in some cases, affect‐regulation has been consistently shown to be the most prevalent function of self‐injury. Furthermore, NSSI carried out for this purpose is often done in private and concealed from others. NSSI may also be performed in peer groups context for the purpose of sensation seeking (e.g., for excitement and exhilaration). To complicate matters, these different functions may be endorsed by the same individual (Klonsky & Muehlenkamp, 2007).

Although NSSI is distinct from suicidal behaviours on the basis of non‐lethal intent (Andover & Gibb, 2010), researchers have consistently found that individuals who engage in NSSI are at increased risk for suicidal behaviour compared with individuals who do not engage in NSSI (Grandclerc, De Labrouhe, Spodenkiewicz, Lachal, & Moro, 2016; Hamza & Willoughby, 2013; Klonsky & Olino, 2008). NSSI is in fact among the strongest predictors of future suicide attempts (Grandclerc et al., 2016). At the same time, only a minority of young adults who engage in NSSI actually engage in suicidal behaviour (Klonsky & Olino, 2008). Identifying individuals at risk of suicide plays a critical role in targeted prevention. Several studies have identified that more frequent engagement in NSSI, using multiple methods and using NSSI to fulfil different psychosocial functions, are associated with higher risk of suicide attempts (Andover & Gibb, 2010; Hamza & Willoughby, 2013). These findings support Joiner's interpersonal theory of suicide that NSSI and suicide are at different ends of the same continuum where greater involvement in NSSI increases an individual's acquired capability for suicide by habituating the individual to fear and pain associated with taking one's own life (Joiner, 2005).

As mentioned, NSSI can present in different ways, varying by method, frequency, severity, function and may be associated with an array of risk factors (childhood maltreatment, difficulties with emotion regulation) and psychopathology (major depressive disorder, borderline personality disorder) or the absence of these factors. The possible combinations of these factors produces a constellation of profiles of NSSI, and this multifariousness accounts for the difficulty in evaluating and managing the condition (Grandclerc et al., 2016). No current treatment for NSSI qualifies as empirically supported or efficacious (Turner, Austin, & Chapman, 2014). A better understanding of this phenomenon and an attempt to identify high‐risk groups would make it more possible to optimize treatment, resource allocation and suicide prevention by identifying self‐injurers who require more extensive treatment.

Reducing the heterogeneity of NSSI presentations by grouping individuals who engage in NSSI into clinically meaningful classes may aid our understanding of this condition and guide treatment plans. A small body of research has attempted to identify typologies of NSSI using a latent class analysis (LCA). LCA is a method for classifying heterogeneous individuals into homogeneous subgroups (Muthén & Muthén, 2000) based on patterns of traits and/or behaviours. One of the earliest studies was conducted by Klonsky and Olino (2008), who identified four groups of self‐injurers. The groups were (1) the ‘Experimental NSSI' group that experimented with NSSI on rare occasions, (2) the ‘Mild NSSI' group that engaged in NSSI more frequently than the Experimental group, (3) the ‘Multiple functions/Anxious NSSI' group that comprised members who used a variety of NSSI methods and endorsed several automatically and socially reinforcing functions of NSSI and (4) the ‘Automatic‐function/Suicidal NSSI' that was characterized by members who almost exclusively cut themselves for automatically reinforcing functions and had more severe clinical symptoms as well as suicidal ideation and behaviours. There have been several attempts to extend and replicate this work by including additional NSSI features (Dhingra, Boduszek, Palmer, & Shevlin, 2015) and other indicators such as childhood adversity (Vaughn, Salas‐Wright, Underwood, & Gochez‐Kerr, 2015) and psychiatric symptoms (Xin et al., 2016). Most of these studies were conducted with college students, and there is a dearth of research in clinical samples.

The current study was conducted in Singapore, a city state located at the tip of the Malay Peninsula in Southeast Asia. NSSI is prevalent among the youth population with a prevalence rate of 60% in a psychiatric sample (Shahwan et al., 2018) and 23% in a survey with 1,095 Singaporeans (Ho, 2019). Local government bodies have been concerned about youth suicide rates and have identified NSSI as an important risk factor (Ang, 2019). The aim of this study was to build upon previous work that had identified typologies of NSSI and examine the extent of generalizability in our local clinical population.

## 2 METHODS

### 2.1 Participants

Participants consisted of 235 outpatients of the Institute of Mental Health (IMH) aged between 14 and 35 years. The age range considered for our sample was based on two local definitions of youth: (1) the Children and Young Persons Act (1993), which defines a young person as 14–16 years, and (2) the National Youth Council, which defines youths as those aged 15–35 years (Singapore|Factsheets|Youthpolicy.org, n.d.). The IMH is Singapore's only tertiary‐care psychiatric hospital. Data of participants who had reported at least one NSSI behaviour within the last year on the Functional Assessment of Self‐Mutilation (FASM) were extracted from a larger study (Shahwan et al., 2018) for the current analysis.

### 2.2 Procedure

Participants were recruited at the child and adult outpatient clinics in IMH between October 2015 and June 2016. Ethics approval was attained from the National Healthcare Group Domain Specific Review Board, and clinicians were informed of the study. Participants either volunteered by responding to posters situated in the outpatient clinics or were referred by clinicians. Patients were screened for intellectual disability as the requirements of the study required considerable insight, Primary 6 (equivalent to 6 years of schooling) reading level and the ability to retrospect. An intellectual disability diagnosis reflected in patient's medical records was a quick way to identify and exclude individuals who were most unlikely to be able to complete the survey adequately. Informed consent was obtained from participants prior to commencement of study procedures. Although a waiver of parental consent was obtained, the study was explained to parents of adolescents less than 21 years of age if they were present. The research officers ensured that the self‐administered questionnaires were completed independently by the participants.

### 2.3 Measures

*FASM* (Lloyd‐Richardson, Perrine, Dierker, & Kelley, 2007): NSSI behaviours were measured using a self‐report checklist designed to assess the frequency of 11 self‐harm behaviours (e.g., cutting, scratching and burning) within the last 12 months. For the purposes of the current study, frequencies of all self‐harm behaviours were summed to indicate the total frequency of NSSIs. Participants were then categorized into three equal proportions based on tertiles: low (one to six times), moderate (seven to 24 times) and high (25 and above). The number of methods endorsed by the participant was also summed to obtain a total number of NSSI methods utilized. The FASM also consists of a checklist of 22 statements measuring the functions of NSSI (e.g., to avoid being with people) rated on a scale of 0 to 3 (0 = *Never*, 1 = *Rarely*, 2 = *Some*, 3 = *Often*). Previous literature has identified a four‐factor structure of the FASM functions checklist: automatic‐negative reinforcement (two items), automatic‐positive reinforcement (three items), social‐negative reinforcement (four items) and social‐positive reinforcement (13 items). In the present study, the Cronbach *α* coefficients were 0.75, 0.65, 0.81 and 0.90, respectively. Participants who selected the response options *Some* or *Often* on any of the items corresponding to that factor were categorized as endorsing NSSI for that function (Lloyd‐Richardson et al., 2007).

*Difficulties in Emotion Regulation Scale (DERS)* (Gratz & Roemer, 2004): The DERS is a 36‐itemself‐report questionnaire that measures six aspects of emotion dysregulation: non‐acceptance of emotional responses (non‐acceptance; six items, *α* = 0.87), difficulties engaging in goal directed behaviour (goals; five items, *α* = 0.84), impulse control difficulties (impulse; six items, α = 0.85), lack of emotional awareness (awareness; six items; *α* = 0.82), limited access to emotion regulation strategies (strategies; eight items, *α* = 0.87) and lack of emotional clarity (clarity; five items, *α* = 0.80). Items are rated on a scale of 1 (*almost never*) to 5 (*almost always*), with higher DERS scores indicating greater difficulties with emotion regulation.

*Childhood Trauma Questionnaire (CTQ)* (Bernstein & Fink, 1998)*:* The CTQ is a 28‐item retrospective self‐report inventory with 25 items assessing the severity of five types of childhood trauma: emotional abuse, physical abuse, sexual abuse, emotional neglect and physical neglect. Responses were rated on a scale of 1 (*never*) to 5 (*very often*). The scores for each subscale ranges from 5 to 25, with higher scores indicating greater experiences of childhood trauma. Guidelines for the classification (none, low, moderate and severe) for each subscale were provided by the manual (Bernstein & Fink, 1998). The CTQ has demonstrated excellent psychometric rigour, with high internal consistency as whole (*α* = 0.97), as well as for each subscale (*α* = 0.81 to 0.95) (Bernstein, Ahluvalia, Pogge, & Handelsman, 1997). With regard to the present study, the Cronbach *α* coefficient as a whole was 0.92, whereas coefficients for the five factors were 0.84, 0.87, 0.93, 0.88 and 0.61, respectively. For the present study and to facilitate analyses and interpretation of results, scores on each subscale were further classified into three groups: none, low/moderate and severe.

*Short Form‐12 (SF‐12)* (Burdine, Felix, Abel, Wiltraut, & Musselman, 2000): The SF‐12 is a 12‐itemhealth‐related quality of life questionnaire that assesses eight health domains: physical functioning, role physical, bodily pain, general health, vitality, social functioning, role emotional and mental health. The number of response options differs across items, ranging from three to six. The SF‐12 can be summarized into two scores: Physical Component Summary (PCS) and Mental Component Summary (MCS). These two scores range from 0 to 100, with increasing scores indicating better health. Based upon the measurement model by Ware, Kosinski, and Keller (1995), the PCS and MCS had Cronbach *α* values of 0.80 and 0.82, respectively.

*Patient Health Questionnaire depression scale (PHQ‐8)* (Kroenke et al., 2009): The PHQ‐8 is an eight‐itemself‐report questionnaire that is designed to assess the severity of depression over the last 2 weeks. Each item is rated on a scale of 0 to 3 (0 = *not at all*, 1 = *several days*, 2 = *more than half the days*, 3 = *nearly every day*). The internal consistency of the PHQ‐8 in the present study was high, with a Cronbach *α* coefficient of 0.89. Based on previous literature, a cut‐off point of larger or equal to 10 on the PHQ‐8 was utilized to define having depression (Kroenke et al., 2009).

### 2.4 Statistical analysis

A LCA was utilized to identify subgroup heterogeneity among individuals who engaged in NSSI in the last year. In the present study, eight latent class indicators of mixed types (two ordinal and six binary) were included in the LCA. The latent class indicators are as follows: (i) frequency of NSSI (ordinal), (ii) length of contemplation before engaging in NSSI (ordinal), (iii) usage of more than three NSSI methods (binary), (iv) suicidal ideation (binary), (v) some/often social‐positive function (binary), (vi) some/often social‐negative function (binary), (vii) some/often automatic‐positive function (binary) and (viii) some/often automatic‐negative function (binary). Four statistical criteria were examined to identify the best fitting model: log likelihood, likelihood ratio chi‐square test, Akaike's information criterion (AIC), Bayesian information criterion (BIC) and entropy values. Lower AIC and BIC values indicate a better class model and good fit, whereas entropy values larger than 0.80 indicate clear delineation of classes (Tein, Coxe, & Cham, 2013). Parsimony and interpretability of the latent class solutions were also considered for the selection of the final model. Following the identification of the final latent class model and assigning participants to the classes based on their posterior probabilities, a multinomial logistic regression was conducted to examine the association between sociodemographic variables (i.e., age, gender, ethnicity and education) and class membership. Thereafter, the effect of significant sociodemographic variables (i.e., age, gender and ethnicity) was adjusted for in the following analyses. A multinomial logistic regressions model was utilized to examine the association between emotion regulation, childhood trauma and class membership. Next, a logistic regression analysis was computed to delineate the association between class membership and likelihood of having depression as measured by the PHQ‐8. Two linear regression models adjusting for significant sociodemographic covariates and the PHQ‐8 were also conducted in order to examine the relationship between class memberships and the physical and mental health scales of the SF‐12. Statistical significance was set at the conventional level of *p* < 0.05, using two‐sided tests. Listwise deletion was implemented in all analyses to handle missing data. All statistical analyses were conducted with Stata version 15.

## 3 RESULTS

### 3.1 Sample characteristics

Out of the 235 participants recruited for the study, the majority were female (*n* = 137; 58.3%) and of Chinese ethnicity (*n* = 160, 68.1%) followed by those of Malay (*n* = 42, 17.9%), Indian (*n* = 23, 9.8%) and other (*n* = 10, 4.3%) ethnicities. The mean age of the sample was 22.0 years (SD = 5.58; range: 14–35). 23.0% (*n* = 54) of the sample were below 18 years of age, 25.1% (*n* = 59) were between 18 and 20 years old, whereas the remaining 51.9% (*n* = 122) were aged 21 and above. All participants had endorsed at least one NSSI method within the past year on the FASM scale.

### 3.2 LCA and identified classes

To determine the optimal solution, the LCA began by extracting one latent class and increasing the number of extracted classes of the solution until no more could be extracted due to nonconvergence. The results indicated that up to three latent classes solution was plausible. All three solutions had non‐significant likelihood ratio test values, indicating that each model did not fit worse than a saturated model. The one‐class solution had a log likelihood of −1365.61, AIC value of 2751.21 and BIC value of 2785.81. Comparatively, the two‐class solution indicated better fit, with lower values of −1253.63, 2549.25 and 2621.90, respectively. However, the entropy value of the two‐class solution was 0.75, indicating that the solution was not optimal with regards to delineation of classes. The three‐class solution was the best fit model due to lower log likelihood, AIC and BIC values of −1223.28, 2510.56 and 2621.27, respectively. In addition, the three‐class solution also possessed an entropy value of 0.8, indicating the clear delineation of classes.

The best fitting LCA model revealed three distinct groups. Class 1 (*n* = 45; 19.2% prevalence) was characterized by low probability of a past suicide attempt (0.09), low probability of using more than three forms of NSSI (0.14), having high (0.67) endorsement of low frequency of NSSI in last year, having high endorsement of ‘none to a few minutes' contemplation before NSSI (0.88) and low endorsement of all reasons/functions of NSSI (probabilities < 0.3). Class 2 (*n* = 74; 31.5% prevalence) had similar characteristics to Class 1 except that individuals in this class had high endorsements on social‐positive use (0.78), automatic‐positive use (0.90), automatic‐negative use (1.00), and moderate endorsement on social‐negative use (0.44). Class 3 was the largest group (*n* = 116; 49.4%). This class was characterized by higher probabilities of a past suicide attempt (0.50) as compared with Classes 1 and 2, higher likelihood of using more than three forms of NSSI (0.80), high probabilities of moderate to high frequencies of NSSI (0.99), contemplating for a few minutes or more (0.73) and high endorsements on all functions for NSSI (0.66 to 0.98). The conditional probabilities of the indicator variables and class membership proportions (based on estimated posterior probabilities) of each class based on the three‐class solution are displayed in Table 1.

**TABLE 1:** Conditional probabilities and class membership proportions (based on estimated posterior probabilities) of the latent class analysis (LCA) solution

		Class 1: Experimental/Mild NSSI		Class 2: Multiple functions NSSI/Low Suicide Ideation		Class 3: Multiple functions NSSI/Possible Suicide Ideation		
		(*n* = 45; 19.2%)		(*n* = 74; 31.5%)		(*n* = 116; 49.4%)		
		Probability (SE)	95% CI	Probability (SE)	95% CI	Probability (SE)	95% CI	
NSSI indicators	Type							
Attempted NSSI for suicide	Binary	0.09 (0.05)	0.03–0.22	0.14 (0.06)	0.06–0.29	0.50 (0.05)	0.40–0.60	
More than three forms of NSSI	Binary	0.14 (0.06)	0.06–0.32	0.05 (0.07)	0.002–0.54	0.80 (0.06)	0.66–0.89	
Frequency of NSSI in last year	Ordinal							
Low (1 to 6)		0.67 (0.08)	0.51–0.80	0.66 (0.09)	0.47–0.81	0.02 (0.03)	0.00–0.45	
Moderate (7 to 24)		0.23 (0.07)	0.13–0.39	0.26 (0.07)	0.15–0.42	0.40 (0.05)	0.30–0.50	
High (25 and above)		0.09 (0.05)	0.03–0.24	0.08 (0.06)	0.02–0.29	0.59 (0.06)	0.47–0.69
Length of contemplation before NSSI	Ordinal						
None		0.50 (0.08)	0.35–0.65	0.36 (0.07)	0.24–0.50	0.27 (0.05)	0.19–0.37
A few minutes		0.38 (0.08)	0.25–0.55	0.40 (0.07)	0.28–0.54	0.29 (0.05)	0.21–0.39
More than a few minutes		0.11 (0.06)	0.04–0.29	0.23 (0.06)	0.14–0.37	0.44 (0.05)	0.34–0.53
Some/often social‐positive use	Binary	0.28 (0.08)	0.16–0.46	0.78 (0.06)	0.64–0.88	0.83 (0.04)	0.74–0.89
Some/often social‐negative use	Binary	0.20 (0.06)	0.11–0.36	0.44 (0.08)	0.30–0.59	0.66 (0.05)	0.57–0.75
Some/often automatic‐positive use	Binary	0.25 (0.07)	0.13–0.41	0.90 (0.07)	0.67–0.98	0.98 (0.02)	0.91–1.00
Some/often automatic‐negative use	Binary	0.15 (0.13)	0.02–0.58	1.00 (0.00)	0.00–1.00	0.89 (0.03)	0.82–0.94

Abbreviation: NSSI, nonsuicidal self‐injury.

### 3.3 Sociodemographic characteristics of latent classes

Sociodemographic characteristics of the latent classes are provided in Table 2. A multinomial logistic regression with class membership as the outcome was conducted to identify the sociodemographic correlates of each latent class. Results are displayed in Table 3. Results revealed that as compared with those who were below 18 years old, individuals who were 21 and above were more likely (OR = 4.74, 95% CI: 1.33–16.91, *p* = 0.02) to be in Class 2 than Class 1. Those of Malay ethnicity were less likely (OR = 0.26, 95% CI: 0.08–0.86, *p* = 0.03) to be in Class 2 than Class 1, when compared with those of Chinese ethnicity. Results also indicated that compared with males, females were more likely to be in Class 3 than in Class 1 (OR = 2.19, 95% CI: 1.01–4.78, *p* = 0.048). As education was not significantly associated with latent class membership, it was removed from further analyses in this paper.

**TABLE 2:** Sociodemographic characteristics of the latent classes (*n* = 235)

	Class 1 (*n* = 45)		Class 2 (*n* = 74)		Class 3 (*n* = 116)	
	*n*	%	*n*	%	*n*	%
Age						
Below 18	12	26.67	9	12.16	33	28.45
18 to 20	11	24.44	15	20.27	33	28.45
21 and above	22	48.89	50	67.57	50	43.10
Gender						
Male	24	53.33	34	45.95	40	34.48
Female	21	46.67	40	54.05	76	65.52
Ethnicity						
Chinese	27	60.00	59	79.73	74	63.79
Malay	9	20.00	6	8.11	27	23.28
Indian	6	13.33	7	9.46	10	8.62
Others	3	6.67	2	2.70	5	4.31
Education						
No formal education/PSLE	4	8.89	3	4.05	14	12.07
Secondary/‘O' level/ ‘N' level	16	35.56	25	33.78	58	50.00
‘A' Level/NITEC/higher NITEC/polytechnic/other diploma	24	53.33	37	50.00	39	33.62
Degree/postgraduate	1	2.22	9	12.16	5	4.31

**TABLE 3:** Results of the multinomial logistic regression examining the association between sociodemographic variables and class membership

	Class 2 vs. Class 1			Class 3 vs. Class 1		
	OR	95% CI	*p*	OR	95% CI	*p*
Age						
Below 18	Ref			Ref		
18 to 20	3.04	0.78–11.92	0.11	2.66	0.83–8.53	0.10
21 and above	**4.74**	1.33–16.91	**0.02**	1.98	0.67–5.83	0.22
Gender						
Male	Ref			Ref		
Female	1.88	0.81–4.36	0.14	**2.19**	0.98–4.69	**0.048**
Ethnicity						
Chinese	Ref			Ref		
Malay	**0.26**	0.08–0.86	**0.03**	0.85	0.33–2.13	0.73
Indian	0.37	0.11–1.32	0.13	0.45	0.14–1.47	0.19
Others	0.38	0.06–2.51	0.32	0.67	0.14–3.20	0.61
Education						
No formal education/PSLE	Ref			Ref		
Secondary/‘O' level/‘N' level	1.65	0.30–9.00	0.56	1.00	0.28–3.63	0.99
‘A' level/NITEC/higher NITEC/polytechnic/other diploma	0.89	0.15–5.34	0.89	0.36	0.09–1.49	0.15
Degree/postgraduate	3.90	0.25–61.14	0.33	1.16	0.09–15.54	0.91

*Note*: Bold print highlights statistically significant odds ratio.

Abbreviations: 95% CI, 95% confidence interval of odds ratio; OR, odds ratio.

### 3.4 Association between class membership, emotion regulation and childhood trauma

A series of logistic and linear regression models were utilized for follow‐up analyses. Means and frequencies of the scales utilized in the follow‐up analyses are presented in Table 4. A multinomial logistic regression adjusting for significant sociodemographic covariates (i.e., age, gender and ethnicity), with class membership as the outcome, and the subscales of the DERS and CTQ as correlates was conducted. Results indicated that higher scores on the impulse (OR = 1.18, 95% CI: 1.02–1.37, *p* = 0.03) and awareness (OR = 1.18, 95% CI: 1.03–1.34, *p* = 0.01) subscales were associated with higher likelihoods of being in Class 3 than in Class 1. Results also revealed that individuals who had low to moderate emotional abuse were less likely (OR = 0.26, 95% CI: 0.07–0.95, *p* = 0.04) than those with no emotional abuse to be in in Class 2 as compared with Class 1. Additionally, individuals who experienced severe physical abuse during childhood were more likely (OR = 13.57, 95% CI: 2.13–86.34, *p* = 0.01) than those who experienced none to be in Class 3 as compared with Class 1. Results from the multinomial logistic regression analyses are presented in Table 5.

**TABLE 4:** Cross tabulation between class membership and means/frequencies on DERS, CTQ, SF‐12 and PHQ

	Class 1 (*n* = 45)		Class 2 (*n* = 74)		Class 3 (*n* = 116)	
	Mean	SD	Mean	SD	Mean	SD
Difficulties in Emotion Regulation Scale						
Non‐acceptance of emotional responses (Non‐acceptance)	13.91	4.19	16.36	5.59	20.62	6.32
Difficulties engaging in goal directed behaviour (Goals)	15.89	4.63	17.05	4.89	19.49	4.58
Impulse control difficulties (Impulse)	15.58	4.47	17.49	5.61	21.71	5.51
Lack of emotional awareness (Awareness)	17.81	5.73	17.43	5.06	18.68	6.04
Limited access to emotion regulation strategies (Strategies)	20.71	6.70	24.65	6.91	29.58	6.98
Lack of emotional clarity (Clarity)	13.78	4.06	14.66	4.44	16.90	4.74
SF‐12						
Physical Component Summary	50.70	8.04	51.90	8.16	49.26	10.06
Mental Component Summary	41.90	10.42	35.24	10.00	28.29	9.63

	Class 1		Class 2		Class 3	
	*n*	%	*n*	%	*n*	%
Childhood Trauma Questionnaire^a^						
Emotional abuse						
None	12	29.27	21	31.34	10	10.10
Low to moderate	23	56.10	32	47.76	41	41.41
Severe	6	14.63	14	20.90	48	48.48
Physical abuse						
None	22	55.00	42	59.15	31	29.81
Low to moderate	11	27.50	16	22.54	27	25.96
Severe	7	17.50	13	18.31	46	44.23
Sexual abuse						
None	34	77.27	50	70.42	62	58.49
Low to moderate	9	20.45	12	16.90	24	22.64
Severe	1	2.27	9	12.68	20	18.87
Emotional neglect						
None	15	37.50	19	29.23	14	13.86
Low to moderate	19	47.50	27	41.54	42	41.58
Severe	6	15.00	19	29.23	45	44.55
Physical neglect						
None	20	45.45	35	49.30	33	30.84
Low to moderate	19	43.18	31	43.66	50	46.73
Severe	5	11.36	5	7.04	24	22.43
Physical Health Questionnaire‐8 items						
Non‐significant to mild depression (<10)	30	66.67	28	37.84	14	12.07
Depression (≥10)	15	33.33	46	62.16	102	87.93

Abbreviations: CTQ, Childhood Trauma Questionnaire; DERS, Difficulties in Emotion Regulation Scale; PHQ, Patient Health Questionnaire; SF‐12, Short Form‐12.

a Due to missing values, total frequency for each subscale of the CTQ may not tally with frequencies of participants each class. Number of missing data for each CTQ subscale is 28, 20, 14, 29, and 13, respectively.

**TABLE 5:** Results from the multinomial logistic regression analyses between class membership, emotion regulation and childhood trauma

	Class 2 vs. Class 1			Class 3 vs. Class 1		
	OR	95% CI	*p*	OR	95% CI	*p*
DERS						
Non‐acceptance	1.05	0.91–1.22	0.48	1.15	0.99–1.33	0.07
Goals	0.95	0.80–1.12	0.53	0.99	0.82–1.19	0.88
Impulse	1.03	0.90–1.17	0.70	**1.18**	1.02–1.37	**0.03**
Awareness	1.02	0.91–1.14	0.78	**1.18**	1.04–1.34	**0.01**
Strategies	1.09	0.93–1.26	0.28	1.08	0.92–1.27	0.33
Clarity	1.01	0.88–1.15	0.88	0.96	0.84–1.11	0.63
CTQ						
Emotional abuse						
None	Ref			Ref		
Low to moderate	**0.26**	0.07–0.95	**0.04**	0.43	0.10–1.97	0.28
Severe	0.18	0.02–1.47	0.11	0.64	0.07–5.85	0.69
Physical abuse						
None	Ref			Ref		
Low to moderate	1.08	0.30–3.90	0.90	1.82	0.46–7.15	0.39
Severe	3.01	0.48–19.01	0.24	**13.57**	2.13–86.37	**0.01**
Sexual abuse						
None	Ref			Ref		
Low to moderate	1.04	0.28–3.95	0.95	2.20	0.58–8.40	0.25
Severe	4.60	0.43–48.95	0.21	2.75	0.25–30.12	0.41
Emotional neglect						
None	Ref			Ref		
Low to moderate	1.49	0.38–5.78	0.57	1.90	0.41–8.89	0.42
Severe	3.24	0.50–20.84	0.22	2.66	0.34–20.69	0.35
Physical neglect						
None	ref			Ref		
Low to moderate	0.51	0.13–1.96	0.33	0.28	0.06–1.26	0.10
Severe	0.28	0.04–2.14	0.22	0.42	0.06–2.96	0.38

*Note*: Bold print highlights statistically significant odds ratio.

Abbreviations: 95% CI, 95% confidence interval of odds ratio; CTQ, Childhood Trauma Questionnaire; DERS, Difficulties in Emotion Regulation Scale; OR, odds ratio.

### 3.5 Association between perceived health, depression and class membership

A logistic regression analysis controlling for the effect of sociodemographic variables (i.e., age, gender and ethnicity) was utilized to examine whether class membership predicted depression based on PHQ‐8 cut‐offs. Results indicated that compared with individuals in Class 1, those in Class 2 (OR = 3.84, 95% CI: 1.67–8.83, *p* = 0.002) and Class 3 (OR = 15.34, 95% CI: 6.46–36.41, *p* < 0.001) were more likely to have depression.

Two separate linear regression models were conducted to examine the association between class membership and the physical component and mental component scales of the SF‐12. These two linear regression models were adjusted for the sociodemographic correlates (i.e., age, gender and ethnicity) and the PHQ‐8 depression scores. Compared with Class 1, membership in Class 3 was associated with lower scores (*β* = −8.04, 95% CI: −11.54 to −4.53, *p* < 0.001) on the mental component scale of the SF‐12. However, when compared with Class 1, membership in Class 2 was not significantly associated with the mental component scale. There was no significant association between class membership and physical component scores. Results of the logistic and linear regression analyses are presented in Table 6.

**TABLE 6:** Logistic and linear regression results of class membership predicting perceived health and depression

Controlled for sociodemographics (i.e., age, gender and ethnicity)	PHQ‐8^a^			SF‐12 Physical Component Score^b^			SF‐12 Mental Component Score^b^		
Variable	OR	95% CI	*p*	*β*	95% CI	*p*	*β*	95% CI	*p*
Class membership									
Class 1	ref			ref					
Class 2	**3.84**	1.67–8.83	**0.002**	1.45	−2.00 to 4.90	0.41	−3.36	−6.90 to 0.19	0.06
Class 3	**15.34**	6.46–36.41	**<0.001**	−0.90	−4.32 to 2.51	0.60	**−8.04**	−11.54 to −4.53	**<0.001**

*Note*: Bold print highlights statistically significant odds ratio or *β* value.

Abbreviations: 95% CI, 95% confidence interval of odds ratio or *β*; *β*, standardized coefficient; OR, odds ratio; PHQ, Patient Health Questionnaire; SF‐12, Short Form‐12.

a Controlled for sociodemographics (i.e., age, gender and ethnicity).

b Controlled for sociodemographics (i.e., age, gender and ethnicity) and PHQ.

## 4 DISCUSSION

Three groups of individuals who engage in NSSI were identified using LCA in this study. The three groups were differentiated by NSSI features, functions, severity and a past history of suicide attempt demonstrating the heterogeneity of self‐injurers in our clinical sample.

### 4.1 Three classes of self‐injurers in psychiatric outpatient sample

Class 1: The first subgroup we identified consisted of 19.2% of young adults with a history of NSSI. This group was congruent to the ‘Experimental/Mild NSSI' group of earlier studies (Case et al., 2019; Klonsky & Olino, 2008). Individuals in this group were characterized by low‐frequency engagement in NSSI in the past year, participation in fewer methods of NSSI (compared with Class 3), low probability of a past suicide attempt and low endorsement of using NSSI to fulfil intrapersonal and interpersonal functions (compared with Classes 2 and 3) suggesting that NSSI was not part of their usual coping repertoire. Interestingly, this group had the shortest length of contemplation before committing the NSSI. The lack of forethought was also found in individuals who committed ‘minor' NSSI compared with moderate or severe injurers in Lloyd‐Richardson's study (Lloyd‐Richardson et al., 2007). We propose that the lack of contemplation may be due to NSSI being of less salience to the mild/experimental group. A study by Selby, Nock, and Kranzler (2014) showed that those with stronger motivation for seeking positive reinforcement from NSSI reported more NSSI thoughts and that NSSI thoughts had a longer duration. It may be that individuals who were likely to endorse multiple functions of NSSI in the two remaining groups were contemplating the reinforcing sensations they would receive from NSSI before acting on their thoughts compared with the mild/experimental group. NSSI among individuals in Class 1 on the other hand may be a spontaneous response to external influence. For instance, several studies have found that participants reported feeling triggered by graphic videos of the behaviour on the web and learning from those who engage in it (Lewis & Seko, 2016; Whitlock, Powers, & Eckenrode, 2006).
Class 2: The second subgroup comprised 31.5% of the sample and was similar to Class 1 in terms of the low‐frequency NSSI engagement, participation in fewer forms of NSSI and low probability of having had a suicide attempt. However, no distinct pattern in length of contemplation was observed. The most striking difference between Class 1 and Class 2 was in the endorsement of functions of self‐harm. Hence, examining the frequency of NSSI alone may lead one to miss out more crucial information. Specifically, Class 2 differs from Class 1 in terms of high endorsements of automatic‐positive use (e.g., to punish yourself), automatic‐negative use (e.g., to stop bad feelings) and social‐positive use (e.g., to get attention). Previous research on the functions of NSSI suggested that intrapersonal functions are most salient in the maintenance of NSSI (Tatnell, Kelada, Hasking, & Martin, 2014). This group thus shares some similarities to Klonsky and Olino's (2008) multiple functions group and Whitlock & Eckenrode's (2008) moderate severity NSSI group in that multiple functions were endorsed but had less severe NSSI features than the most severe class that also demonstrate suicidality. We termed this group ‘Multiple functions NSSI/Low Suicide Ideation'.
Class 3: The third subgroup comprised 49.4% of the sample. This group was characterized by high‐frequency engagement in NSSI, participation in more than three forms of NSSI, high endorsement of all the functions of NSSI, that is all three functions endorsed by Class 2 and in addition, the social‐negative use of NSSI (e.g., to avoid doing something unpleasant you do not want to do). Class 3 was also distinct from Classes 2 and 1 in that participants were more likely to have a longer period of contemplation before committing NSSI and had a higher probability of having suicidal intent when engaging in self‐harm. This group differed from Klonsky and Olino's only suicidal group that tended to endorse only automatic functions and was more similar to Somer et al.'s, (2015) and Bracken‐Minor, McDevitt‐Murphy, and Parra's (2012) most severe group that was characterized by high frequencies of multiple methods of NSSI, high endorsement of suicide risk factors and high levels of psychopathology. However, like Klonsky and Olino's automatic functions/suicidal group, there was a longer response latency for this group suggesting a deliberate, premeditated strategy to regulate negative emotions. Endorsement of social functions was highest for this group suggesting that individuals may have interpersonal skill deficits that could increase the vulnerability for turning to NSSI within an interpersonal context (Muehlenkamp, Brausch, Quigley, & Whitlock, 2013). We speculate that the distribution of participants which was highest in this group was due to sample differences as earlier studies demonstrating the lowest distribution in this group was conducted in college student samples. We termed this group, ‘Multiple functions NSSI/Possible Suicide Ideation'.

### 4.2 Correlates of three classes of self‐injurious behaviours

A higher distribution of participants aged 21 and above but lower distribution of participants below 18 years and who were of Malay ethnicity (compared with Chinese) were found to be in Class 2 compared with Class 1. In regard to age, NSSI is most prevalent during middle adolescence (Barrocas, Hankin, Young, & Abela, 2012). A longitudinal study of adolescents in urban and rural schools in China showed that the majority of individuals who self‐injure showed declining NSSI from Grades 10–12 (ages 15–17) (Barrocas, Giletta, Hankin, Prinstein, & Abela, 2014) but some adolescents continue to engage in high levels of NSSI in a more chronic manner. This phenomenon could account for the age patterning in our data among the classes. The reason for the lower proportion of Malays in Class 2 compared with Class 1 is unclear. Further research on local ethnic differences is needed to shed light on ethnic differences between the classes. It was of note that Class 3 had twice the number of females as males suggesting that there may be important differences in the development and course of NSSI by gender worthy of future analyses. For example, earlier studies have found that childhood maltreatment independently increased the odds of NSSI for females, and this relationship was explained by the higher tendency for females towards self‐blame (Swannell et al., 2012).

There was a trend of worsening of scores for depression (PHQ‐9) with Class 3 having the poorest scores. After controlling for the PHQ‐8 scores, Class 3 also had significantly lower mental component scores of the SF‐12 scores than Class 1. This was in line with earlier LCA studies that demonstrated greater psychopathology and symptoms scores with more severe NSSI symptoms. Results of the Klonsky and Olino's study showed that their multifunction/anxiety group and automatic/suicide group had higher depression scores compared with the experimental/mild groups. Hamza and Willoughby and Bracken‐Minor et al. also observed worst symptoms scores in the classes with NSSI of greater severity and had previous suicide attempts.

Class 3 had significantly higher rates of physical abuse falling in the severe classification compared with Class 1. Vaughn et al. (2015) in their study examining latent clusters in NSSI based on childhood adversity found that their severe abuse class evinced high levels of psychiatric/psychological issues. Our earlier study (Peh et al., 2017) had demonstrated that there was a dose–response relationship between the exposure to childhood maltreatment and self‐harm frequency and that emotion dysregulation mediated this relationship. Building on our earlier study, we observed that two particular components of difficulties in emotion regulation difficulties were associated with Class 3: lack of emotional awareness and a tendency to lose control in the face of negative emotions. This is congruent with past literature that emotion regulation difficulties are positively associated with NSSI frequency and diversity (Peterson, Chen, Karver, & Labouliere, 2019).

Putting it all together, early exposure to violent or abusive environments may disrupt healthy emotion regulation and socioemotional skills (Trickett, Negriff, Ji, & Peckins, 2011) that may predispose a child with a tendency towards experiencing high levels of negative affectivity (Marshall, Tilton‐Weaver, & Stattin, 2013). Structural equation modelling studies have shown that emotion dysregulation was indirectly associated with NSSI frequency via internalizing symptoms (Kranzler, Fehling, Anestis, & Selby, 2016). When faced with stress or negative affectivity such as anger, guilt and fear, individuals with difficulties in emotion regulation may be less able to tolerate these difficult emotions and internalize their distress. Internalizing symptoms may then increase an individual's likelihood in engaging in NSSI. Ecological momentary assessment studies have demonstrated that high arousal negative states (e.g., frustration and feeling overwhelmed) often precedes NSSI and feelings of relief and calm follow rapidly after the act―supporting an emotion regulatory function of NSSI (Armey, Schatten, Haradhvala, & Miller, 2015).

Interestingly, Class 1 had higher probability of exposure to emotional abuse than Class 2. Although this relationship contradicts the trend of our other findings between abuse and NSSI, it is possible that the higher rates of emotional abuse rates are associated with other conditions or behaviours not measured in this study.

### 4.3 Clinical implications

Results from the LCA are suggestive that different NSSI subgroups may have different treatment indications. Class 1 presents with self‐injurious behaviours that are experimental/mild and may benefit from treatment that monitors NSSI risk but may not require a more comprehensive treatment approach versus those in Class 3 who present with multiple risk factors and psychopathology. Class 2 demonstrates coping with negative emotions through NSSI that may maintain the behaviour, and this group may benefit from learning diverse coping strategies. Although there has yet to be a forerunner for effective treatment of repetitive self‐harm, common elements across treatment models that have shown promise for individuals such as those in Class 3 who struggle with emotional awareness, impulsivity and interpersonal deficits include emotion regulation and distress tolerance skills to build greater acceptance of uncomfortable emotions (Linehan, 1993), mindfulness practice to reduce impulsivity (Dixon et al., 2019) and skills training to strengthen interpersonal bonding and family relationships such as communication training, problem‐solving and/or conflict management (Hetrick, Robinson, Spittal, & Carter, 2016; Turner et al., 2014). Given that individuals are at highest risk shortly after hospital discharge, early intensive treatment may be necessary during this high‐risk period to minimize this risk following discharge (Glenn, Franklin, & Nock, 2015).

### 4.4 Limitations and future directions

There were a number of limitations in this study. First, the study relied on self‐reported data. Although we aimed to minimize socially desirable responses through assurance of anonymity, subjective interpretation of responses could influence the responses. Next, some items such as that of the CTQ require retrospective recollection and may be subject to recall biases. Third, the study was cross‐sectional in nature, precluding establishment of directionality and causality of the indicators and correlates, and the stability of the classes over time. Future studies shedding light on stability and movement across the classes over time as well as prediction of treatment outcome for each class will be valuable. Fourth, history of past suicide attempt was assessed using only a single item on the FASM. An assessment of the extent of tissue damage and suicide risk including acquired capability of suicide (i.e., lowered fear of death and increased physical pain tolerance) using a structured tool may provide a better measure of suicide risk and distinguish between those with suicide desire and those with acquired capability to provide an even finer grain distinction of individuals falling in Class 3, our largest group. Lastly, identifying latent classes entails some error because classes are not perfectly discrete. Nevertheless, our three classes have substantial overlap with that of earlier studies demonstrating generalizability of the classes across geographical locations, cultures and populations.

## 5 CONCLUSION

Our LCA demonstrates that even in a clinical population, NSSI is not homogeneous with increasing class membership signifying increasing severity of NSSI, psychosocial skill deficits and clinical symptomatology. Clinicians working with clients with NSSI should evaluate presenting NSSI features (method and frequency), the functions served by NSSI, emotion regulation difficulties, trauma history and clinical symptoms to better understand an individual client's risk profile and assess suicide risk. Although a clear forerunner in the treatment of NSSI is presently lacking, profiling patterns of NSSI may be a potentially useful step in providing a nuanced examination among individuals with varying histories of NSSI in order to aid clinicians in their case conceptualisation and in prescribing tailored intervention. Intervention research that shed light on treatment strategies based on NSSI subtype is recommended to improve treatment protocols and guidelines for NSSI.

## CONFLICT OF INTEREST

The authors declare that they have no conflict of interest.

## AUTHOR CONTRIBUTIONS

SS designed the study, led the recruitment and wrote the first draft of the article. LJH conducted the data analysis whereas EA reviewed the analysis; both statisticians also reviewed the final draft of the manuscript. ZYJ, RS and TWL recruited participants and reviewed the final draft of the manuscript. CSA secured grant for the study and together with OSH and BG enabled access to participants. These clinicians also reviewed the final draft of the manuscript. MS provided guidance and supervision to the study team from the conception of the study till the study ended and provided advice on early drafts of the manuscript.

## ETHICS APPROVAL AND CONSENT TO PARTICIPATE

Ethical approval for the study was granted by the National Healthcare Group Domain Specific Review Board (DSRB No. 2014/01099). Written informed consent was obtained from all individual participants.
